# Proline improves switchgrass growth and development by reduced lignin biosynthesis

**DOI:** 10.1038/s41598-019-56575-9

**Published:** 2019-12-27

**Authors:** Cong Guan, Hui-Fang Cen, Xin Cui, Dan-Yang Tian, Dimiru Tadesse, Yun-Wei Zhang

**Affiliations:** 1 College of Grassland Science and Technology, China Agricultural University, Beijing, China; 2 Beijing Key Laboratory for Grassland Science, China Agricultural University, Beijing, China; 3 National Energy R&D Center for Biomass (NECB), Beijing, China; 4 Beijing Sure Academy of Biosciences, Beijing, China; 5 Department of Plant and Soil Sciences, Institute for Agricultural Bioscience, Oklahoma State University, Oklahoma, OK USA

**Keywords:** Molecular engineering in plants, Plant morphogenesis

## Abstract

Transgenic switchgrass overexpressing *Lolium perenne* L. delta1-pyrroline 5-carboxylate synthase (*LpP5CS*) in group I (TG4 and TG6 line) and group II (TG1 and TG2 line) had significant P5CS and ProDH enzyme activities, with group I plants (TG4 and TG6) having higher P5CS and lower ProDH enzyme activity, while group II plants had higher ProDH and lower P5CS enzyme activity. We found group II transgenic plants showed stunted growth, and the changed proline content in overexpressing transgenic plants may influence the growth and development in switchgrass. RNA-seq analysis showed that KEGG enrichment included phenylpropanoid biosynthesis pathway among group I, group II and WT plants, and the expression levels of genes related to lignin biosynthesis were significantly up-regulated in group II. We also found that lignin content in group II transgenic plants was higher than that in group I and WT plants, suggesting that increased lignin content may suppress switchgrass growth and development. This study uncover that proline can appropriately reduce lignin biosynthesis to improve switchgrass growth and development. Therefore, appropriate reduction in lignin content and increase in biomass are important for bioenergy crop to lower processing costs for biomass fermentation-derived fuels.

## Introduction

Proline is known as a compatible solute (osmolyte) and a scavenger of reactive oxygen species (ROS) providing protection against oxidative damage^1^. In addition to its well established role in coping with environmental stress, proline also plays an increasingly significant role in plant development. It was reported that proline might have certain regulatory functions during protein synthesis and may act as a signaling molecule during plant development^1^, and proline plays an important role in plant growth and life cycle, such as regulating cyclin genes and affecting general protein synthesis^2^. Additionally, proline may also play critical roles in cellular metabolism both as a component of proteins and as a free amino acid^3^. Many genes are involved in the proline synthesis and degradation pathways. In higher plants, Δ1-pyrroline-5-carboxylate synthetase (P5CS) and proline dehydrogenase (ProDH) are rate-limiting enzymes during the synthesis and degradation of proline respectively. Glutamate is reduced to pyrroline-5-carboxylate (P5C) by P5CS, which is then converted into proline by Δ1-pyrroline-5-carboxylate reductase (P5CR)^1^. On the other hand, proline degradation takes place in the mitochondria, where it is catalyzed into glutamate by ProDH and P5C dehydrogenase (P5CDH)^4^.

Proline metabolism occupies a central place in plant metabolism and is connected to other pathways through both ornithine and glutamate. It is connected to the pentose phosphate pathway and the TCA cycle as a way of moving the reductants and buffering the redox status of the chloroplast^4,5^. In the proline biosynthesis pathway, the consumption of the reductants (NADPH) buffers the redox status of the chloroplast, which is linked with the pentose phosphate pathway. In the pentose phosphate pathway, glucose-6-phosphate (G-6-P) is reduced to ribulose-5-phosphate (Ru-5-P) by the rate-limiting enzymes glucose 6-phosphate dehydrogenase (G6PDH) and 6-phosphogluconate dehydrogenase (6PGDH), and concurrently consumes NADP^+^ to generate NADPH in this process^6^. In plants, transketolase (TK) plays a role in Calvin cycle while in non-photosynthetic organisms it connects the phosphate pentose pathway and glycolysis for generating NADPH^7^. In addition, proline degradation contributes carbon for TCA cycle^4^. The pentose phosphate pathway and Calvin cycle provide erythrose-4-phosphate (E4P) which together with phosphoenolpyruvate acts as precursor for phenylalanine biosynthesis through the shikimic acid pathway^5^. Phenylalanine, as the precursor amino acid for lignin biosynthesis, is essential for secondary cell wall biosynthesis^8^. In most plants, lignin is mainly composed of hydroxyphenyl (H), guaiacyl (G) and syringyl (S) monolignol subunits that are derived from *p*-coumaryl, coniferyl and sinapyl monolignols, respectively. Several enzymes are required for monolignol biosynthesis, including phenylalanine ammonia-lyase (PAL), caffeic acid 3-Omethyltransferase (COMT), caffeoyl-CoA O-methyltransferase (CCoAOMT), hydroxycinnamoyl-CoA reductase (CCR), cinnamyl alcohol dehydrogenase (CAD)^9^. A number of reports have showed that lignin content is reduced by down-regulating the expression of monolignol biosynthesis related genes in plants^10^. Many studies had showed that lignin content had the negative relationship with the growth and development of plants. Transgenic aspen with suppressed *Pt4CL1* expression exhibited up to a 45% reduction of lignin and a 15% increase in cellulose, but leaf, root, and stem growth were substantially enhanced^11^. Additionally, compared with WT plants, *AtLOV1* transgenic switchgrass plants had higher total lignin content, delayed flowering time and less aboveground biomass^12^. Besides, a number of studies suggested that the most severe deficiency of lignin showed stunted growth in plants^13^. However, many studies also showed that lignin can be reduced without reducing yield or fitness^14,15^.

Switchgrass (*Panicum virgatum* L.) is a perennial C4 grass native to North America, considered as a potential dedicated bioenergy crop due to its high biomass production and tolerance on marginal land^9^. We found that overexpression plants showed two different phenotypes, the two groups transgenic plants had different P5CS and ProDH enzyme activities, as well as proline content. To shed light on potential changes in growth and development, we analyzed the RNA-seq data. The results showed that phenylpropanoid biosynthesis pathway was significantly up-regulated in the group II plants compared with the group I and WT plants. In particular, lignin content in group II transgenic plants was higher than that in group I and WT plants, suggesting that proline affects switchgrass growth and development by coordination with lignin biosynthesis.

## Results

### P5CS and ProDH enzyme activities in the two groups of transgenic plants

**Figure 1:**
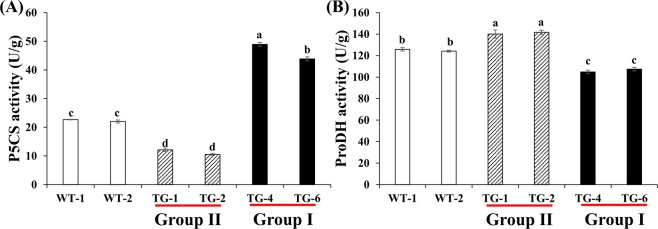
Proline metabolism related enzyme activities. P5CS (**A**) and ProDH (**B**) activities in transgenic and WT plants. Value are mean ± SE (*n* = 6). The significance of treatments was tested at the *P* < 0.05 level (one way ANOVA, Dunnett’s test).

P5CS and ProDH are the rate-limiting enzymes in the proline synthesis and degradation pathway, respectively^1^. P5CS and ProDH activities showed significant differences among the group I (TG4 and TG6), group II (TG1 and TG2) and WT plants. P5CS activity was lower in group II and higher in group I as compared to the wild type (Fig. 1A) where the P5CS activity in group I plants was 4.1 and 2.1 fold greater than group II and WT plants respectively. On the contrary, ProDH activity was the highest in the group II plants (142 U/g), followed by the WT (124 U/g), and lowest in the group I plants (102 U/g) (Fig. 1B). Additionally, group II overexpression lines showed relatively lower proline content, and the proline content of group I transgenic plants was higher compared with WT plants^16^. Combined with the proline levels, these results suggested that proline synthesis is reduced and proline degradation is enhanced in the group II plants compared with the WT plants, and while in group I, there is an increased proline synthesis and decreased proline degradation.

### Coenzyme II NADP(H) contents in the two groups of transgenic plants

**Figure 2:**
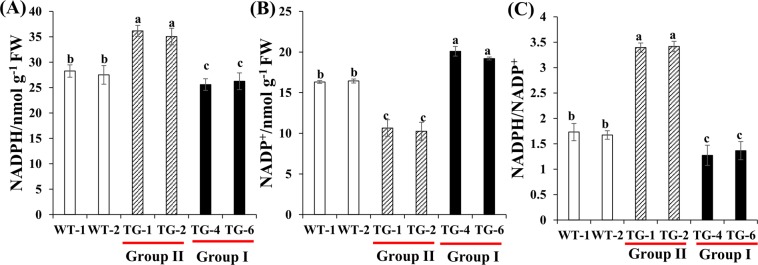
Coenzyme II NADP(H) contents. Coenzyme II NADP (**A**), NADP^+^ (**B**) content and NADPH/NADP^+^ (**C**) ration in the transgenic plants overexpressing *LpP5CS*. Value are mean ± SE (*n* = 6). The significance of treatments was tested at the *P* < 0.05 level (one way ANOVA, Dunnett’s test).

Group II transgenic plants contain lower proline content, higher ProDH and lower P5CS enzyme activity compared with the WT plants. NADP^+^ and NADPH are generated during the synthesis and degradation of proline, respectively; proline synthesis consumes NADPH to regenerate NADP^+^ in the chloroplast, which indicates the NADPH/NADP^+^ ratio is an important index for proline synthesis^17^. The coenzyme II NADP(H) content and NADPH/NADP^+^ ratio was significantly different between the two groups of overexpression lines and WT plants (Fig. 2). The coenzyme II NADP^+^ content was the highest in group I plants, followed by the WT, and lowest in group II plants. Conversely, coenzyme II NADPH was the highest in group II plants, 2.0- and 2.6-fold greater than that in WT and group I plants, respectively. Additionally, the NADPH/NADP^+^ ratio was higher in group II plants and lower the group I plants compared with WT plants. These results suggested that the change in P5CS and ProDH enzyme activities may result in the difference of coenzyme II NADP(H) contents in the group I, group II and WT plants, and simultaneously changing the proline level.

### Morphological characteristic in the two groups of transgenic plants

**Figure 3:**
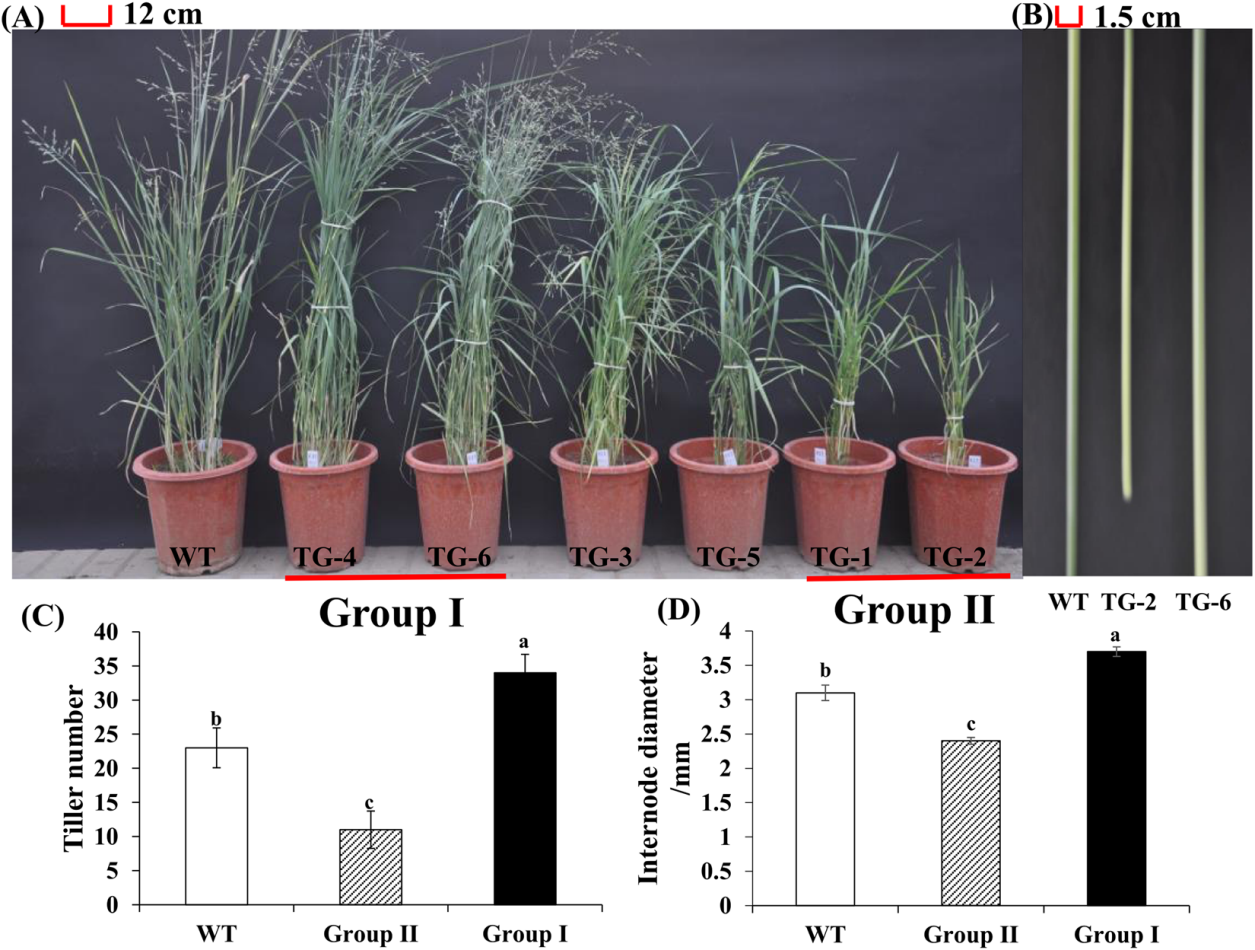
Morphological characterization of transgenic switchgrass plants overexpressing *LpP5CS*. Plant phenotypes (**A**); Stem morphologies (**B**); Tiller number (**C**); Internode diameter (**D**). Value are mean ± SE (*n* = 3). The significance of treatments was tested at the *P* < 0.05 level (one way ANOVA, Dunnett’s test).

As shown in the Fig. 3A,B, group II plants exhibited more stunted growth, such as slender stem and fewer tillers. Compared to the WT plants, transgenic plants in group II had a 52% and 23% reduction in tiller number and internode diameter, respectively; while transgenic plants in group I had a 48% and 19% increase in tiller number and internode diameter, respectively (Fig. 3C,D).

### RNA-seq analysis

**Figure 4:**
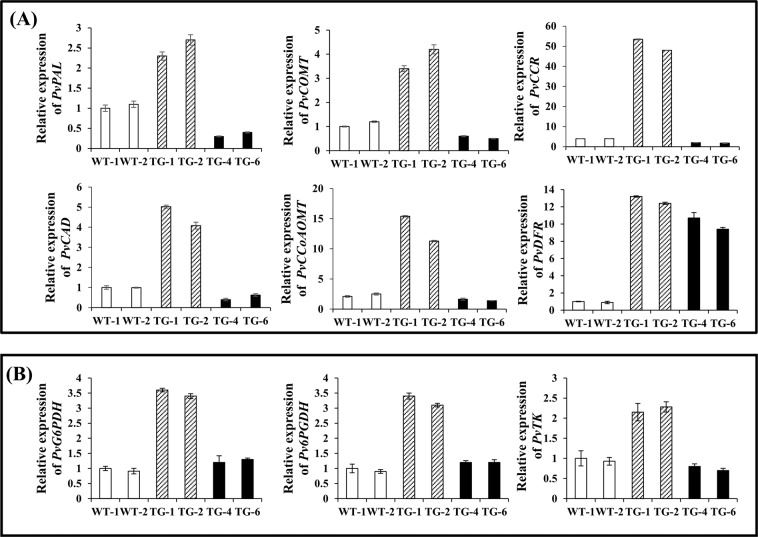
Expression levels analysis of phenylpropanoid biosynthesis (**A**) and pentose phosphate pathway related genes (**B**) in transgenic plants. Switchgrass *Ubq1* was used as the reference for normalization. Value are mean ± SE (*n* = 6).

In the RNA-seq data, the top 20 obviously enriched KEGG pathways are shown in Supplementary Fig. S1. In group II and group I transgenic plants, these significantly enriched pathways include “Phenylpropanoid biosynthesis (KO: zma00940)”, “Stilbenoid, diarylheptanoid and gingerol biosynthesis (KO: zma00945)”, “Cyanoamino acid metabolism (KO: zma00460)”, “Flavonoid biosynthesis (KO: zma00941)” and “Biosynthesis of secondary metabolites (KO: zma00564)”. Additionally, 13 pathways were significantly enriched in the group II and WT plants. These include “Phenylpropanoid biosynthesis (KO: zma00940)”, “Biosynthesis of secondary metabolites (KO: zma01110)”, “Glutathione metabolism (KO: zma00480)”, “Cysteine and methionine metabolism (KO: zma00270)”, “Stilbenoid, diarylheptanoid and gingerol biosynthesis (KO: zma00945)”, “Pentose phosphate pathway (KO: zma00030)”, “Cyanoamino acid metabolism (KO: zma00460)”, “Flavonoid biosynthesis (KO: zma00941)”, “Selenocompound metabolism (KO: zma00450)”, “Nitrogen metabolism (KO: zma00910)”, “Biosynthesis of amino acids (KO: zma01230)”, “Carbon metabolism (KO: zma01200)” and “Alanine, aspartate and glutamate metabolism (KO: zma00250)”. Finally, ten pathways were found significantly enriched which include “Fatty acid degradation (KO: zma00071)”, “Tyrosine metabolism (KO: zma00350)”, “Selenocompound metabolism (KO: zma00450)”, “Isoquinoline alkaloid biosynthesis (KO: zma00950)”, “Starch and sucrose metabolism (KO: zma00500)”, “Biosynthesis of secondary metabolites (KO: zma01110)”, “Tropane, piperidine and pyridine alkaloid biosynthesis (KO: zma00960)”, “Fatty acid metabolism (KO: zma01212)”, “Peroxisome (KO: zma04146)”, “Carbon metabolism (KO: zma01200)”. Besides, the enriched KEGG pathways also included the photosynthesis and circadian rhythm-plant pathways (Supplementary Fig. S2), suggesting that proline metabolism affects the photosynthesis and circadian rhythm in switchgrass. Notably, “Phenylpropanoid biosynthesis” pathway was significantly enriched (*P*-value < 0.05). The expression levels of those DEGs including *PvPAL*, *PvCOMT*, *PvCCoAOMT*, *PvCYP98A3*, *PvCCR*, *PvCAD, PvG6PDH*, *Pv6PGDH* and *PvTK* (Fig. 4), were all significantly up-regulated in the group II plants. Especially, the expression level of *PvCCR* in group II was 12-fold greater than in the WT. In summary, the metabolic network of enriched pathways included arginne and proline metabolism, flavonoid biosynthesis, phenylpropanoid biosynthesis and pentose phosphate pathway, indicating that proline metabolism is involved in those metabolism pathways in switchgrass (Supplementary Fig. S3).

### Lignin monomer compositions in the two groups of transgenic plants

**Figure 5:**
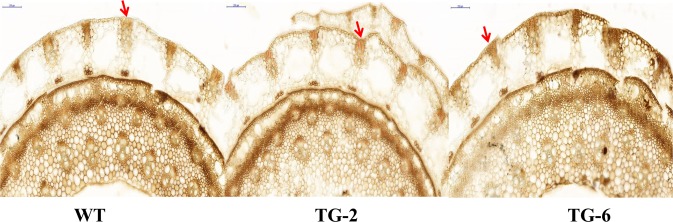
Lignin histological staining by Mäule. The basal internode1 at R1 stage were used to make 50 μm thick cross sections for staining. Scale bars = 200 μm.

In the data of RNA-seq, the expression level of differentially expressed genes (DEGs) involved in the phenylpropanoid biosynthesis were significantly up-regulated in the group II plants compared with the group I and WT plants. Moreover, the lignin monomer composition in the two groups of transgenic and WT plants (Table 1), showed a relatively lower guaiacyl (G) and syringyl (S) contents, as well as hydroxyphnyl (H) content in group I plants. While group II transgenic plants showed relatively higher guaiacyl (G) and syringyl (S) contents, as well as lower hydroxyphenyl (H) content compared with the WT plants. Notably, the ratio of S/G was lower in the group I plants, and the lignin content (S + G + H monomer content) showed a significant difference between the two groups of transgenic and WT plants. Lignin content was lower in the group I plants and higher in the group II plants compared with the WT. In addition, S monomer content of the stem from TG2, TG6 and WT at the R1 stage was evaluated after staining with the Mäule reagent, we found that the S monomer content (Mäule staining) was clearly reduced in the stem of TG6 (Fig. 5). These results suggest that proline metabolism affects lignin biosynthesis in switchgrass.

**Table 1:** Lignin composition in the group I and group II transgenic switchgrass.

	Total lignin (μmol/g CWR)	H lignin (μmol/g CWR)	G lignin (μmol/g CWR)	S lignin (μmol/g CWR)	S/G
WT	279.06 ± 1.98b	7.04 ± 0.12ab	154.05 ± 0.32ab	117.97 ± 0.96a	0.77a
Group I-TG	250.03 ± 7.25c	7.83 ± 0.28a	151.51 ± 3.04b	90.68 ± 1.82b	0.6b
Group II-TG	290.23 ± 2.53a	6.86 ± 0.04b	162.04 ± 0.54a	121.34 ± 1.22a	0.75a

^*^Internode 3 were collected from each transgenic and WT plants grown in soil for 5 months, six tillers were collected for each replicate. Total lignin content = S + G + H monomer content. Values are means ± SE (*n* = 6). The significance of treatments was tested at the *P* < 0.05 level (one way ANOVA, Dunnett’s test).

## Discussion

P5CS activity was lower in group II plants, which could led to the decreased proline accumulation. In the present study, KEGG enrichment included pentose phosphate pathway (PPP), fatty acid synthesis and phenylalanine biosynthesis pathway, and at the same time, NADP^+^ and NADPH content showed a significant difference between the two groups of transgenic and WT plants. NADP^+^ and NADPH are generated during the synthesis and degradation of proline, respectively^18^. The cycling of proline substrate is coupled to maintaining the NADP^+^/NADPH ratio via the pentose phosphate pathway^19^, and PPP provides NADPH for fatty acid synthesis in plastids^6^. So it seems that proline degradation was accelerated in the group II transgenic plants, which led to an increase in the NADPH content.

It was reported that antisense *AtP5CS* transgenic Arabidopsis led to proline depletion and abnormal leaf formation^20^. Down-regulation of proline biosynthesis genes, on the other hand, resulted in growth defects^21^, indicating the importance of proline for plant development. In our study, the stems were significantly thicker in group I transgenic plants and thinner in group II plants, when compared with the WT plants (Fig. 3D). Furthermore, as the results of the RNA-seq analysis indicated, the enriched KEGG pathways also include the photosynthesis and circadian rhythm-plant pathways (Supplementary Fig. S2). This leads us to conclude that proline may play important roles in plant growth and development.

**Figure 6:**
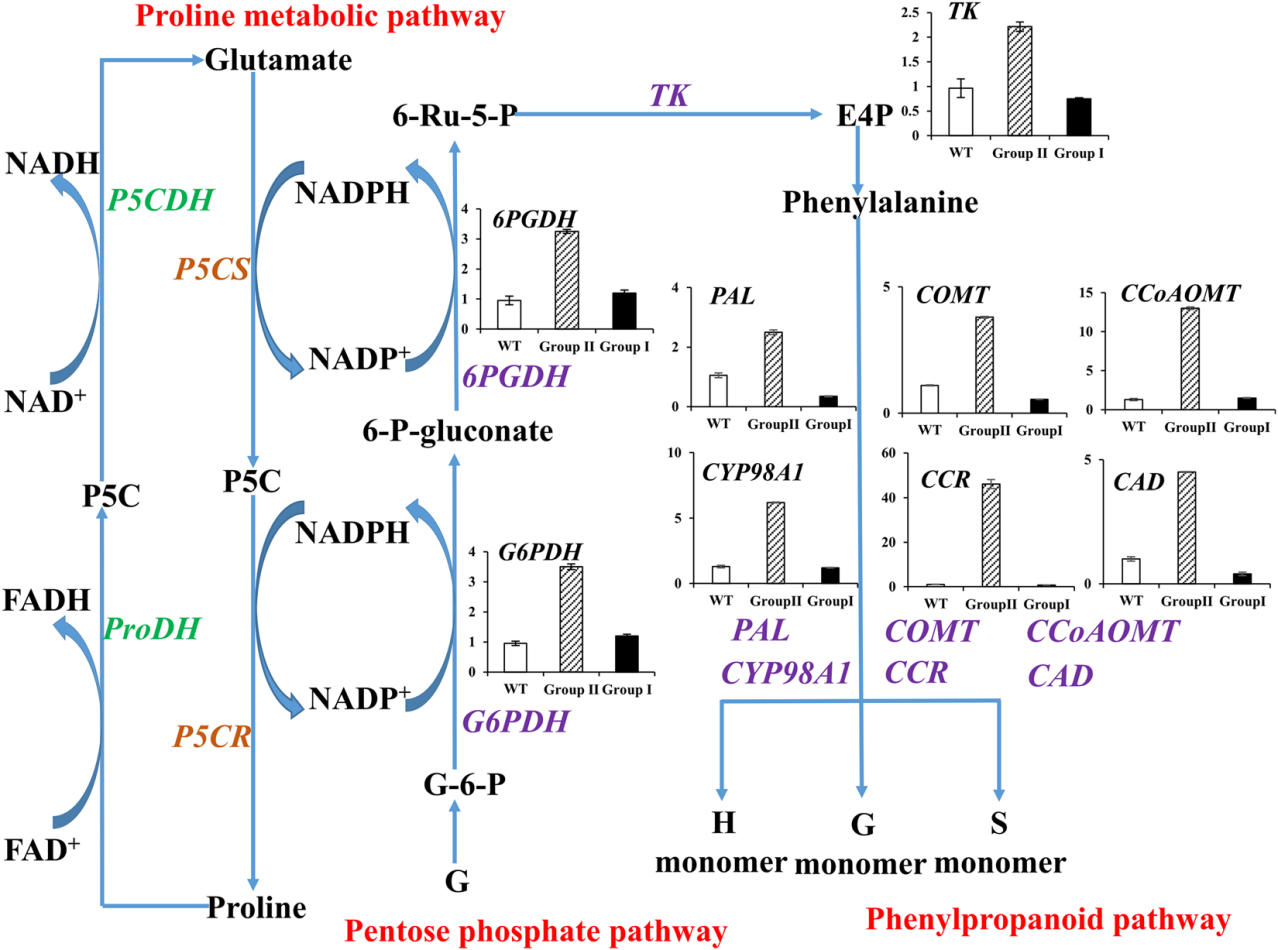
Metabolic network diagram between phenylpropanoid biosynthesis pathway, pentose phosphate pathway and proline metabolic pathway.

Proline metabolism is connected to the pentose phosphate pathway (PPP) and the TCA cycle, and the PPP and Calvin cycle provide erythrose-4-phosphate (E4P) which together with phosphoenolpyruvate acts as a precursor for phenylalanine biosynthesis through the shikimic acid pathway^5^. In our result of RNA-seq, the enriched KEGG pathways were including arginine and proline metabolism, flavonoid biosynthesis, phenylpropanoid biosynthesis and pentose phosphate pathway (Supplementary Fig. S3). To precisely understand the relationship between proline metabolism and lignin biosynthesis in switchgrass, we summarized the metabolic network that exists between the phenylpropanoid biosynthesis pathway, pentose phosphate pathway and proline metabolic pathway in Fig. 6. In these metabolism pathways, *PvG6PDH* and *Pv6PGDH* are genes encoding key enzymes involved in the pentose phosphate pathway; *PvPAL*, *PvCOMT*, *PvCCoAOMT*, *PvCCR* and *PvCAD* are the genes encoding for important enzymes in lignin biosynthesis, and transketolase (TK) participates in the Calvin cycle. The proline biosynthesis pathway is linked with the pentose phosphate pathway through consuming the reductants (NADPH), while the pentose phosphate pathway and the Calvin cycle provide erythrose-4-phosphate (E4P) for phenylalanine biosynthesis. Thus, proline metabolism pathway is coordinated with phenylpropanoid biosynthesis pathways in switchgrass. In particular, our results showed that the lignin content (S + G + H monomer content) was lower in group I plants and higher in group II plants compared with the WT. So, reducing proline biosynthesis may induce the increase of lignin content in group II transgenic plants. Many studies showed that lignin content affected plant growth and development. Suppressing *Pt4CL1* expression can reduce the lignin content and enhance the growth of leaf, root, and stem in aspen^11^, and RNA interference of *Pv4CL1* lead to a reduction in lignin content with uncompromised biomass yields^15^. Additionally, overexpressing *AtLOV1* gene increased total lignin content, delayed flowering time and reduce the aboveground biomass in switchgrass^12^. Besides, extreme lignin deficiency can also contribute to stunted growth in plants^13^. In our result, group II transgenic plants which had lower proline and higher lignin content exhibited more stunted growth. Thus, we speculate that proline affects switchgrass growth and development by coordination with lignin biosynthesis.

In conclusion, unlike the previous studies that proline plays an important role in regulating cyclin genes and affecting general protein synthesis to improve plant growth and development. Our study showed that lacking proline could result in an increased lignin content, and leading to the stunted growth in switchgrass. However, the regulatory mechanism how proline plays roles in plant growth and development is still unclear, and understanding the molecular mechanism will help us to prime candidates in crop genetic engineering for improving the biomass of plants. Moreover, appropriate reduction in lignin content and increase in biomass is one important strategy of bioenergy crop to lower processing costs for biomass fermentation-derived fuels.

## Materials and Methods

### Plant materials and growth conditions

Switchgrass callus generated from mature seeds (Alamo) was transformed with a pCAMBIA 1301-*LpP5CS* overexpression cassette using the Agrobacterium-mediated transformation method^16^. Group I consists of two independent overexpression lines (TG4 and TG6) while group II contains TG1 and TG2. The two groups of transgenic switchgrass plants were grown in the greenhouse under a 16 h light/8 h dark photoperiod at 25 ± 2 °C. The switchgrass plants were used for the measurement of tiller number and stem diameter at reproductive stage (R3), and each line comprises three biological samples.

### P5CS and ProDH enzyme activities

P5CS and ProDH activities in the transgenic and control plants were assayed based on the protocal^22,23^. Briefly, frozen switchgrass leaves (0.1 g) were ground in liquid nitrogen and mixed with 1 mL of extraction buffer (0.1 M Tris–HCl, pH 7.2, 10 mM MgCl2, 10 mM 2-mercaptoethanol, and 1 mM PMSF). The homogenate was centrifuged at 12,000 g for 20 min at 4 °C and the P5CS activity was calculated. Switchgrass leaves (0.1 g) were homogenized into powder and placed in 0.5 mL extraction buffer (100 mM sodium phosphate, 1 mM cysteine, and 0.1 mM EDTA [pH 8.0]). After centrifugation at 12,000 g for 10 min at 4 °C, the supernatant was used to measure ProDH activity. Each assay included two technical with three biological replicates.

### Coenzyme II NADP (H) contents

The coenzyme II NADP(H) contents in the two groups of overexpression lines and WT plants were measured according to the protocol^24,25^ with some modifications. Briefly, swithgrass leaves (0.1 g) were ground in liquid nitrogen and homogenized with 0.1 M HCl (for NADP assay) or 0.1 M NaOH (for NADPH assay). Samples were heated at 95 °C for 2 min and cooled in an ice bath. After samples were centrifuged for 10 min at 4 °C, and the supernatants were used for coenzyme assay. Assays were performed using a reaction volume of 200 mL on 96-well plates, and absorbance was measured at 565 nm from 0 to 30 min after the start of the reaction. Two technical and three biological replicates of each transgenic line were performed in each experiment.

### RNA-seq analysis

Total RNA was extracted from transgenic and WT leaves at the E5 stage, and RNA samples included three groups: TG1 and TG2 lines (group II), TG4 and TG6 lines (group I), and WT1 and WT2 plants. The RNA-seq data were downloaded from NCBI, the SRA number is SRP130275^16^. The paired-end reads were aligned to the reference genome (https://phytozome.jgi.doe.gov/pz/portal.html#!info?alias=Org_Pvirgatum_er) using TopHat v2.0.12^26^. The HTSeq v0.6.1^27^ and DEGSeq R package^28^ were used to count the reads numbers mapped to each gene and determine if any differential expression exists. The KOBAS software was performed to test the statistical enrichment of differential expression genes in KEGG pathways.

### Quantitative real-time RT-PCR analysis

Total RNA from the leaves of the transgenic and WT plants at elongation stage (E5) was isolated by the TRIzol reagent method (Invitrogen, Carlsbad, CA, USA). Quantitative real-time RT-PCR analysis was performed following the description^17^, the data were normalized using the level of switchgrass *Ubq* transcripts (GenBank accession no. FL955474.1), and the relative expression levels of genes were calculated using the 2^−△△CT^ method^29^. Two biological with three technical replicates of each line were performed in each experiment. The primers used for qRT-PCR are listed in Table S1.

### Lignin monomer composition

Lignin monomer levels of stems from the two groups of transgenic and WT lines at the reproductive stage (R1) were measured following the description^30^. Three technical replicates of each line were performed in the experiment.

### Histology and microscopy

The internode 1 (I1) of stem collected from transgenic and control plants at the R1 stage was used to make 50 μm thick cross sections^31^. This was followed by Mäule staining as described^32^. Images were taken under an Olympus BX-51 compound microscope, and the data were analyzed using Image-Pro Plus 6.0.

### Statistical analysis

Triplicate samples were collected for each transgenic and WT lines. Data from each trait were subjected to analysis of variance (ANOVA). The significance of the difference between treatments was tested at the *P* < 0.05 level. Standard errors are provided in tables and figures as appropriate. All the statistical analyses were performed using the SPSS package (SPSS 20.0, IBM Company, USA).

## Supplementary information

Supplementary Information
