# Supplementary material for: Proline improves switchgrass growth and development by reduced lignin biosynthesis
Source: Sci Rep. 2019 Dec 27;9:20117. doi: 10.1038/s41598-019-56575-9 (PMC6934488; doi:10.1038/s41598-019-56575-9)
Supplement: Supplementary file 1 — Supplementary Information [file 41598_2019_56575_MOESM1_ESM.docx]

Article title: **Proline improves switchgrass growth and development by reduced lignin biosynthesis**

Cong Guan^1^, Hui-Fang Cen^1^, Xin Cui^1^, Dan-Yang Tian^1^, Dimiru Tadesse^5^ and Yun-Wei Zhang ^1, 2, 3, 4*^

**The following Supporting Information is available for this article:**

**Figure S1** KEGG enrichments of the annotated DEGs. Group II VS Group I (a); Group II VS WT (b); Group I VS W (c). The left Y-axis indicates the KEGG pathway. The X-axis indicates the Rich factor. A high q-value is represented by blue, and a low q-value is represented by red.

**
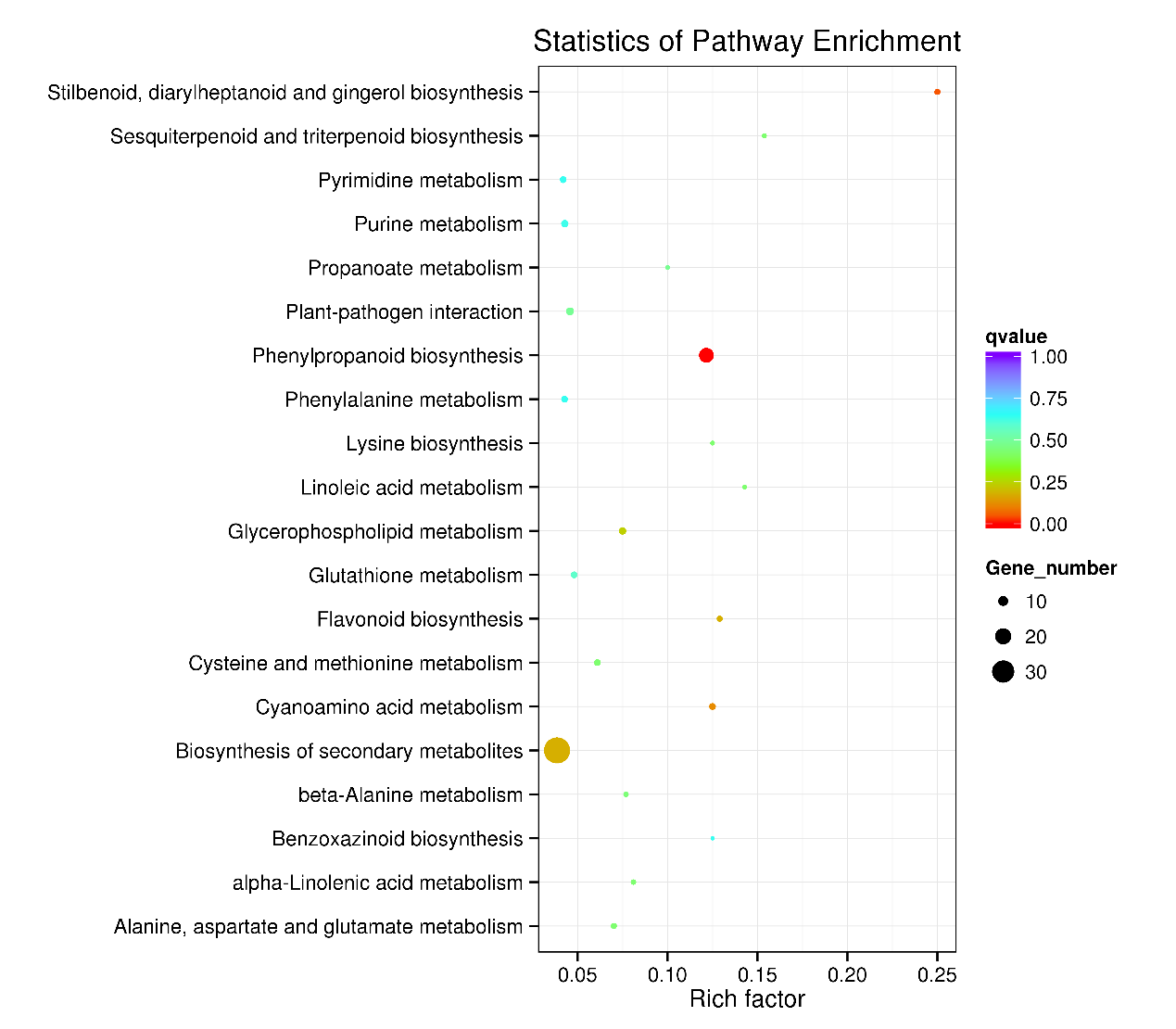
**

**a Group II VS Group I**

**
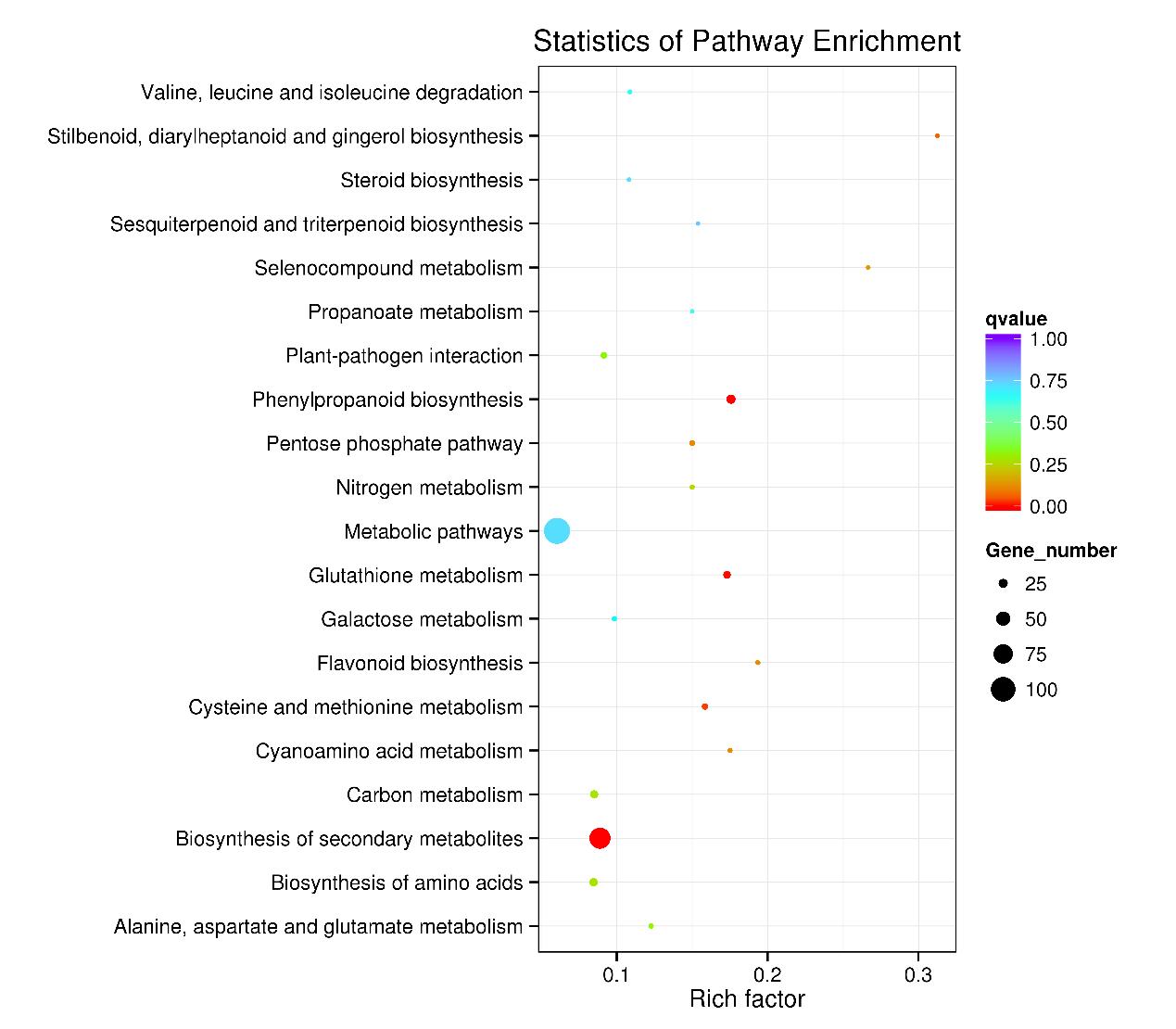
**

**b Group II VS WT**


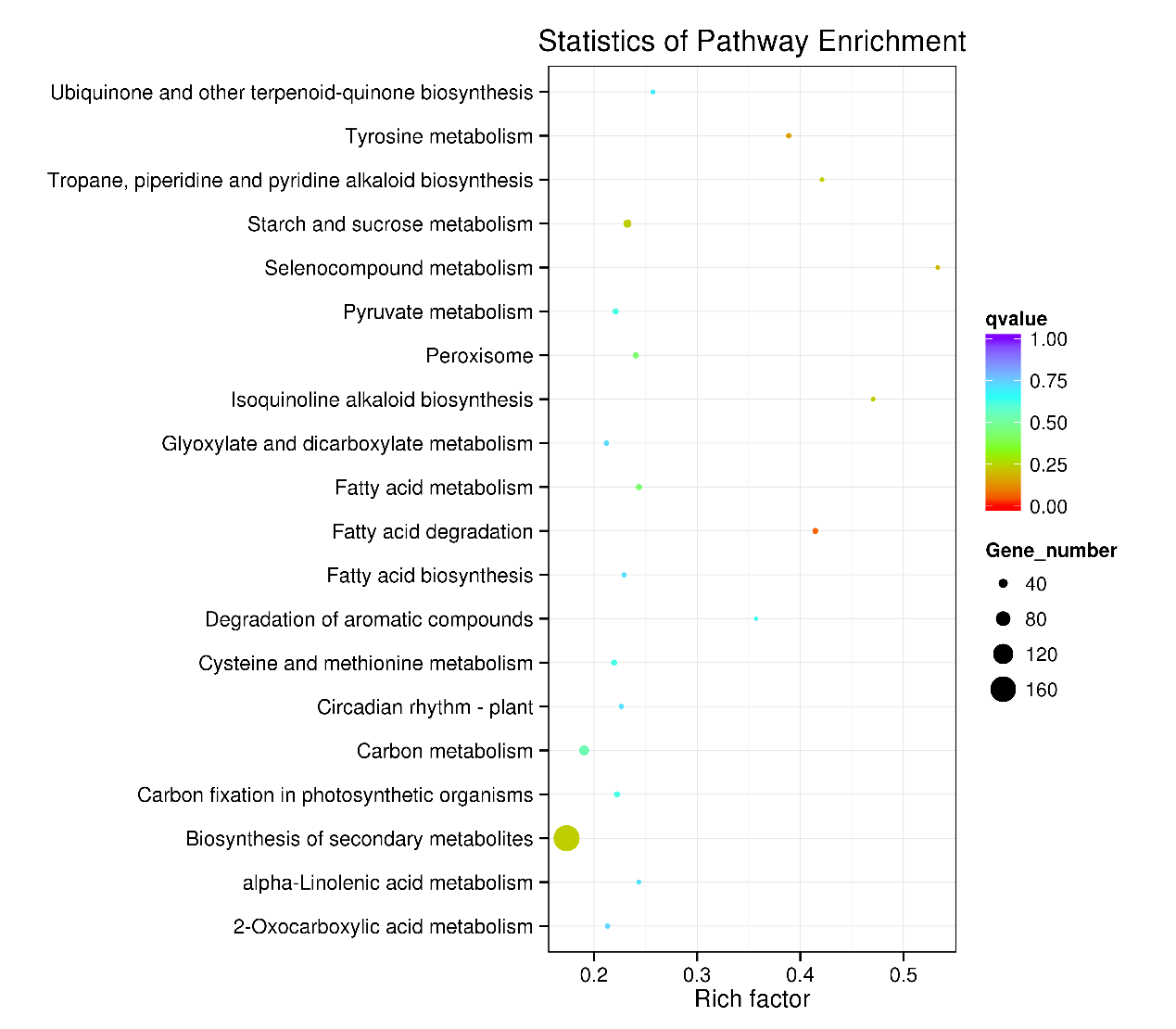


**c Group I VS WT**

**Figure S2** The pathways of photosynthesis (a) and circadian rhythm-plant (b) enriched by KEGG analysis. The KEGG pathway database is gained from Kanehisa laboratories^1^.


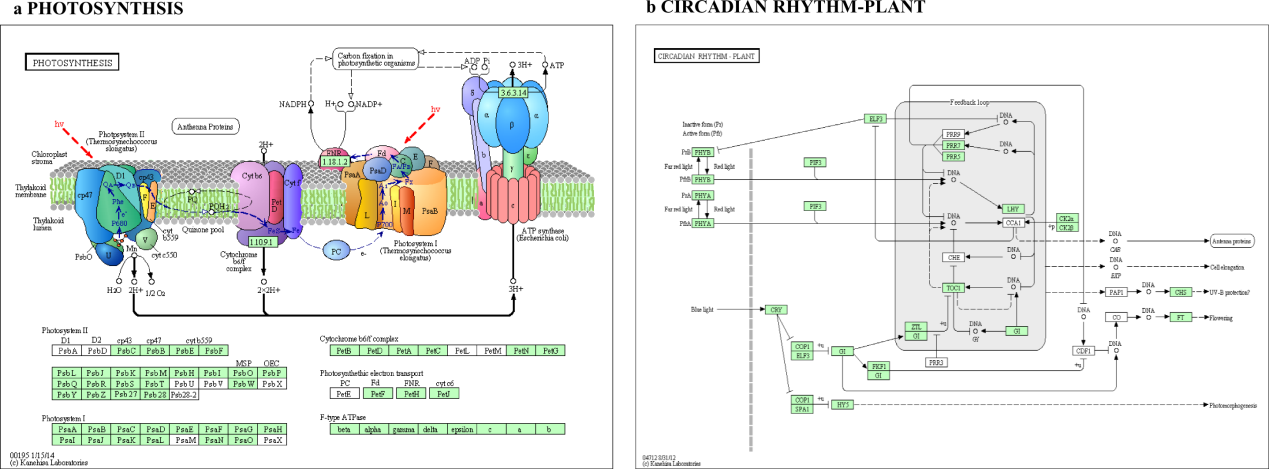


**Figure S3.** The pathways of arginne and proline metabolism (a), pentose phosphate pathway (b), phenylpropanoid biosynthesis (c) and flavonoid biosynthesis (d) enriched by KEGG analysis. The KEGG pathway database is gained from Kanehisa laboratories^1^.

**a**


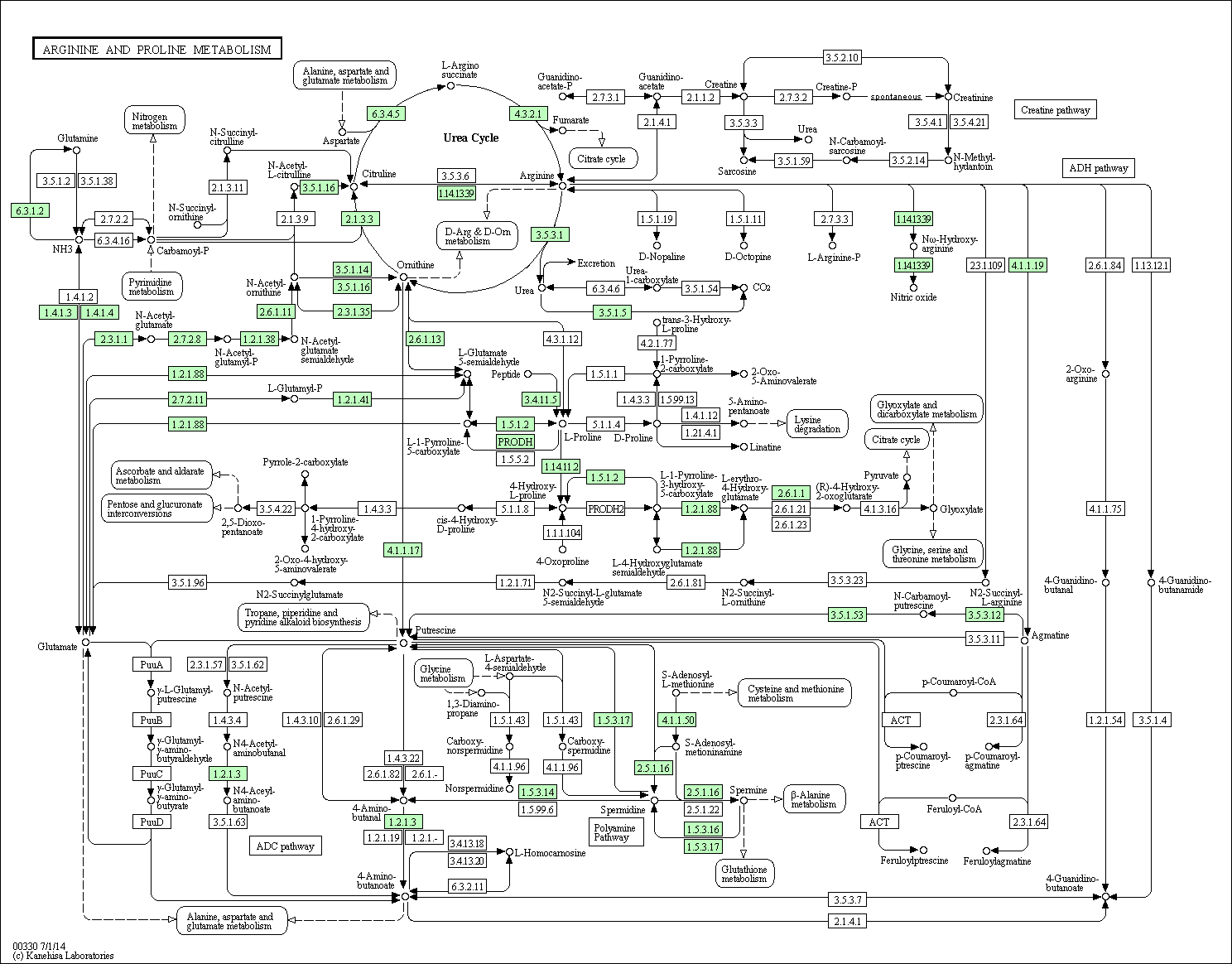


**b**


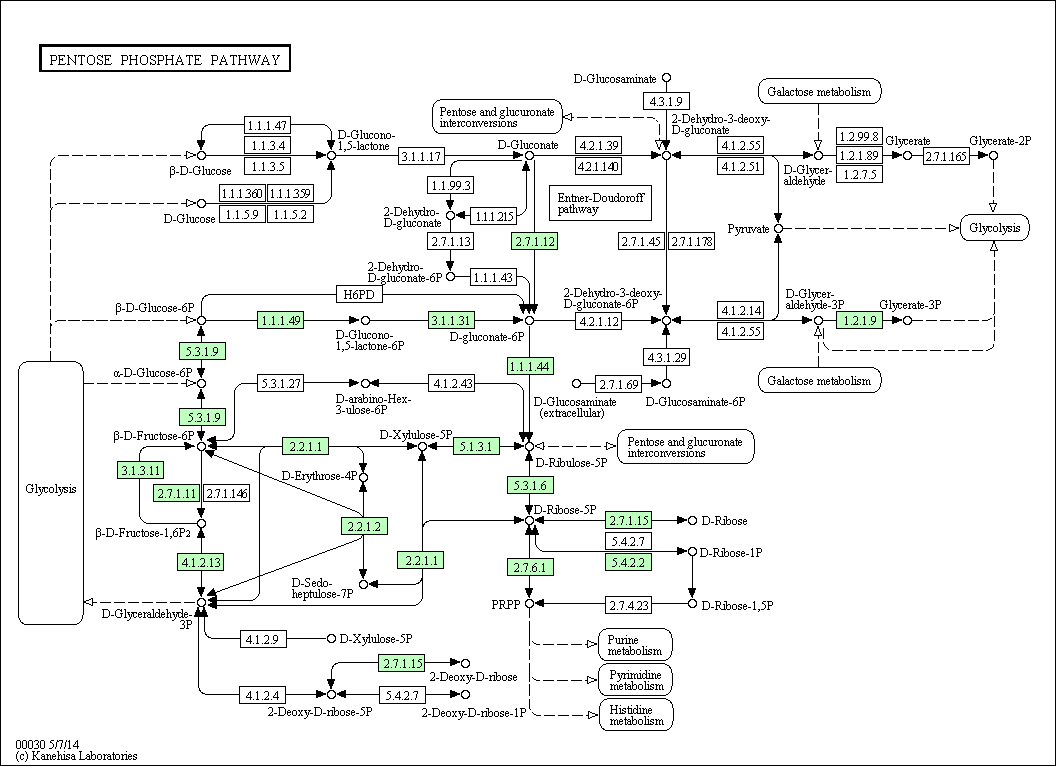


**c**


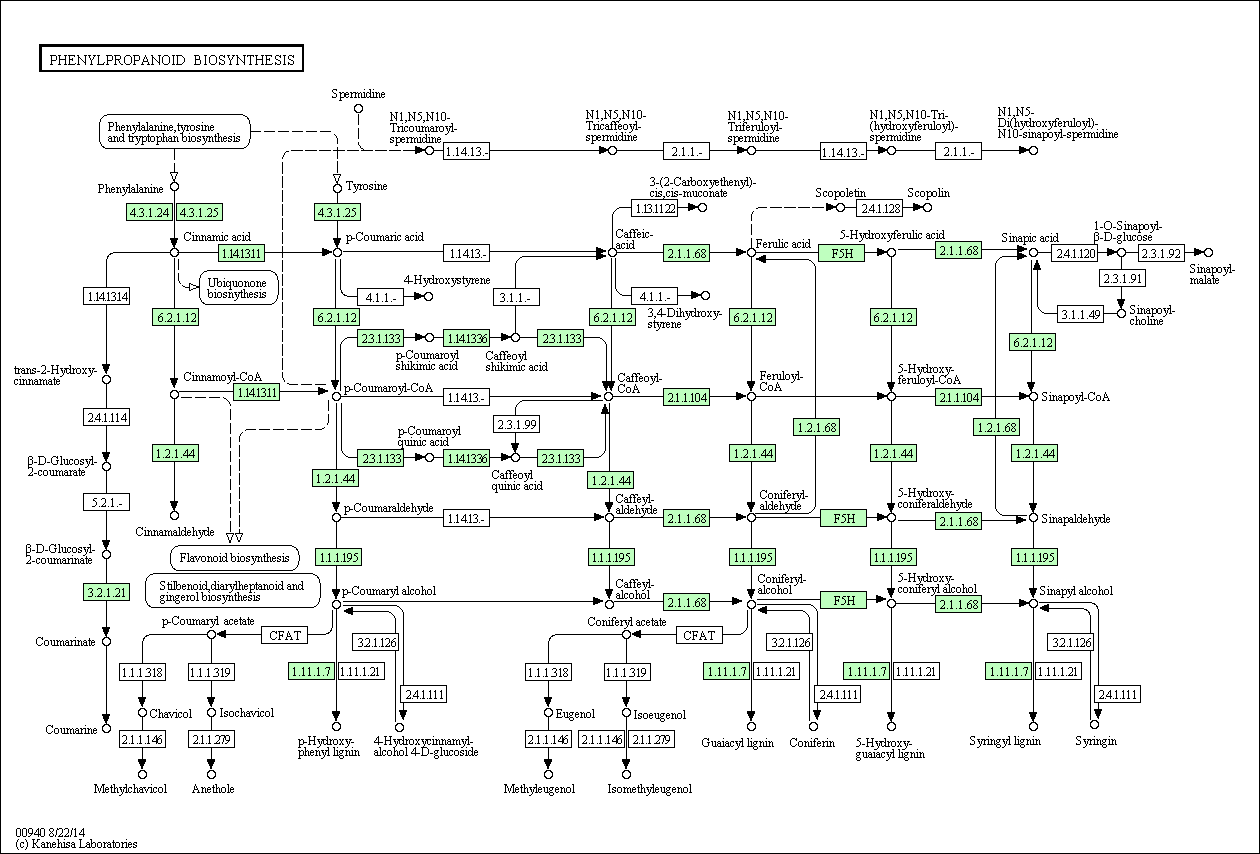


**d**


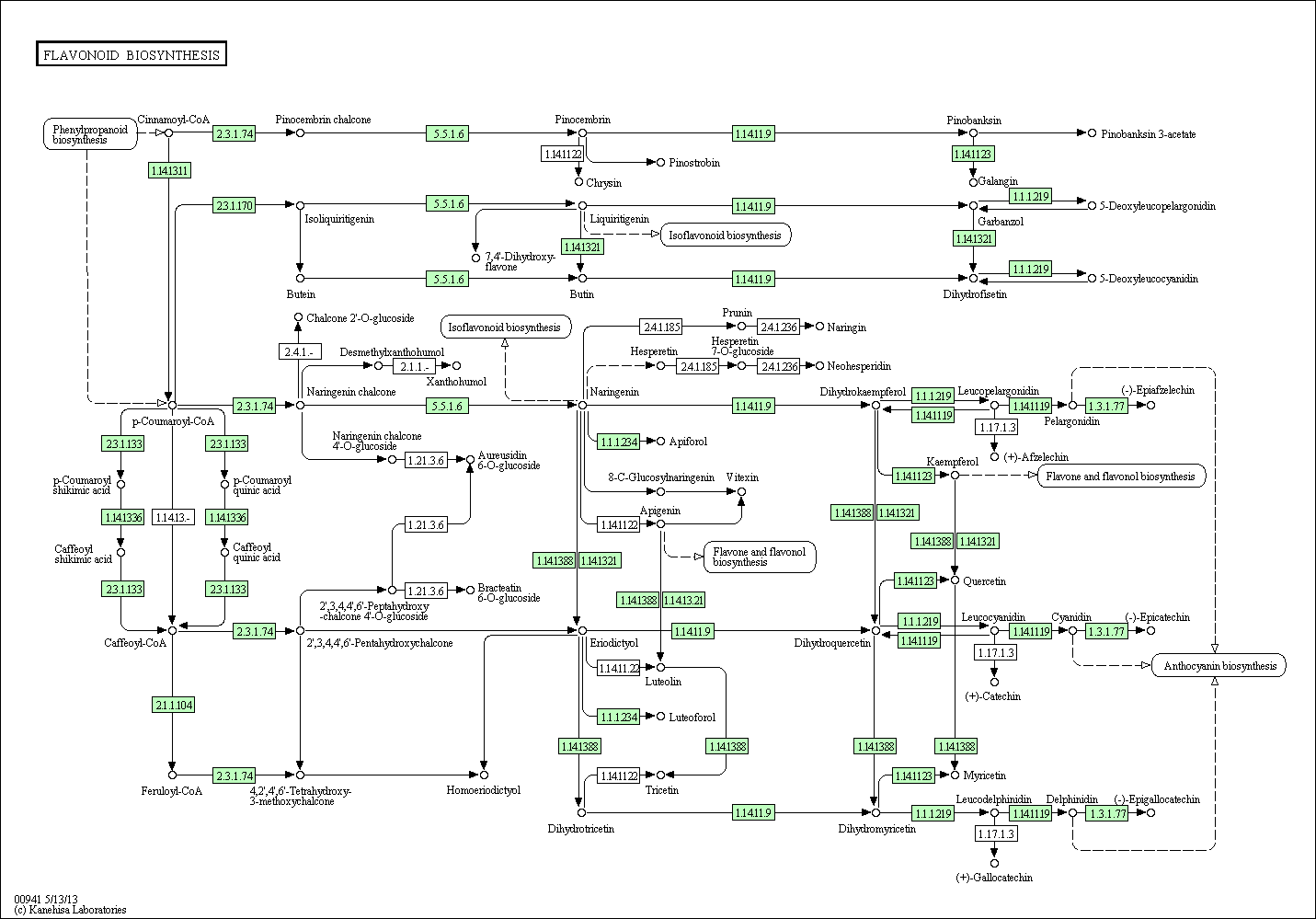


**References**

1. Kanehisa, M. and Goto, S. KEGG: Kyoto Encyclopedia of Genes and Genomes. *Nucleic Acids Res* **28**, 27-30 (2000).

| Name/Accession no. | Sequence (5’-3’) | Purpose |
| --- | --- | --- |
| *PvPAL* | F1: CATACCCGCTGTACAGGTTC | Quantitative expression of |
| (Pavir.Gb00641.1) | R1: AACACCTTGTTCACCTCCTC | *PvPAL* |
| *PvCCR*  (Pavir.Aa00132.1)  *PvCAD*  (Pavir.Ga02655.1)  *PvCCoAOMT*  (Pavir.Fa00353.1)  *PvCOMT*  (Pavir.Ga02435.1)  *PvCYP98A3*  (Pavir. J02261.1)  *PvG6PDH*  (Pavir.Ia03470.1)  *Pv6PGDH*  (Pavir.Ea03982.1)  *PvTK*  (Pavir.Ea03881.1) | F2: ATGGCGCCACGAATGAA  R2: CAAGTCTTGCTAGGGACATCAG  F3: TGGTCCTACAACGACGTCTA  R3: GATCGGCACCACGAACTT  F4: GCCGCCCGACTCCAATCAT  R4: GTCTCGACATTTACGCATGAAA  F5: CGGCATCCCGTTCAACAAGG  R5: TGGTTCTTCATGCCCTCGTTGA  F6: ATGAATGCAAACGGTGAGATTG  R6: AACTCAGCAACAGAGAGAGATG  F7: TGGATTGAGGTGCTGGATTG  R7: CCTTCGAGCATCTAGGTTTCTT  F8: CCAAGAGCTCAGAGAAAGGATG  R8: TTGATGCGATCGAGGAAGATG  F9: CTGCTTATCCTGACACCCTTTAT  R9: GAAACAGTGCCGTGGTCTAT | Quantitative expression of *PvCCR*  Quantitative expression of *PvCAD*  Quantitative expression of *PvCCoAOMT*  Quantitative expression of  *PvCOMT*  Quantitative expression of  *PvCYP98A3*  Quantitative expression of  *PvG6PDG*  Quantitative expression of  *Pv6PGDH*  Quantitative expression of  *PvTK* |

**Table S1** Primers used in this study
